# Hypoxia in MASLD: a spatial determinant of the pathogenesis

**DOI:** 10.1016/j.molmed.2025.12.008

**Published:** 2026-01-15

**Authors:** Isabel Fuster-Martínez, Vanesa Bernal-Monterde, Guillaume Bidault, José M. Arbonés-Mainar, Antonio Vidal-Puig

**Affiliations:** 1 Institute of Metabolic Science, University of Cambridge, Cambridge, UK; 2 Health Investigation Institute Aragón (IIS-A), Zaragoza, Spain; 3 Adipocyte and Fat Biology Laboratory (AdipoFat), Translational Research Unit, Miguel Servet University Hospital, Zaragoza, Spain; 4 Gastroenterology Department, Miguel Servet University Hospital, Zaragoza, Spain; 5 Instituto Aragonés de Ciencias de la Salud (IACS), Zaragoza, Spain; 6 CIBER Fisiopatología Obesidad y Nutrición (CIBERObn), Instituto Salud Carlos III, Madrid, Spain; 7 Victor Phillip Dahdaleh Heart and Lung Research Institute, University of Cambridge, Cambridge, UK; 8 Centro de Investigation Principe Felipe, Valencia, Spain

## Abstract

The liver has a unique microarchitecture, with hepatic sinusoids receiving blood from the portal vein and hepatic artery and draining into the central vein. This flow establishes an oxygen gradient along the sinusoids critical for defining the liver zonation. In metabolic dysfunction-associated steatotic liver disease (MASLD), fat accumulation and fibrosis disrupt this architecture, contributing to localised hypoxia. Mounting evidence implicates hypoxia in MASLD, including the activation of canonical hypoxia sensors such as hypoxia-inducible factors. Moreover, chronic intermittent hypoxia, characteristic of obstructive sleep apnoea (OSA), is epidemiologically and mechanistically associated with MASLD progression. This review examines the intrahepatic oxygen dynamics, the interplay between OSA and MASLD, and molecular responses to hypoxia, proposing intrahepatic hypoxia as a spatial determinant of liver injury.

## Intrahepatic oxygen levels in MASLD

Oxygen (O_2_) availability has been a major determinant of cellular metabolism since the Great Oxidation Event ~2.4 billion years ago, driving the emergence of adaptive mechanisms that allow cells to function across a wide range of O_2_ tensions. In modern physiology, reduced O_2_ availability (hypoxia) elicits metabolic adaptations that favour anaerobic pathways [1]. The liver is uniquely organised to exploit such metabolic flexibility, with a specialised vascular architecture that establishes a physiological O_2_ gradient along the hepatic sinusoids, underpinning metabolic zonation [2]. In **metabolic dysfunction**–**associated steatotic liver disease (MASLD** (see Glossary), the most prevalent chronic liver condition [3], the liver’s finely tuned organisation becomes progressively disrupted by hepatocellular injury, inflammation, and fibrotic remodelling. This impairs the diffusion of O_2_ within the liver and leads to increased hypoxia.

Recent advances in spatial multi-omics technologies enable a more comprehensive characterisation of hypoxia-induced cellular responses. When combined with direct or indirect spatial measurements of O_2_ levels, these methods could unravel how hypoxia sensitises hepatocytes and non-parenchymal cells to disease progression. This review summarises the current evidence supporting the hypothesis that intrahepatic O_2_ levels are a key factor in the pathogenesis of MASLD by creating zonated vulnerability along the hepatic sinusoids.

### Oxygen in liver zonation and pathogenesis

**Figure 1:**
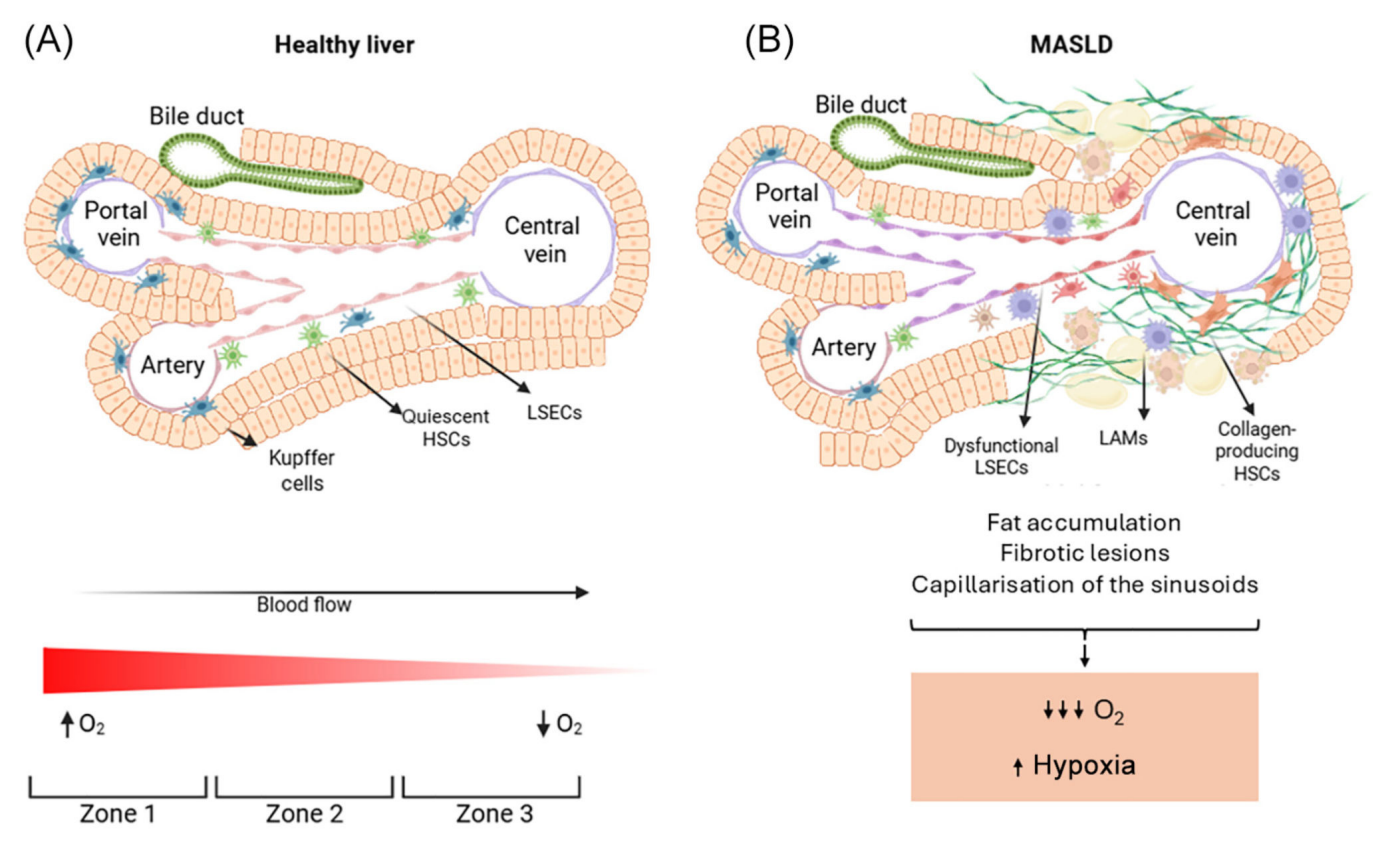
Physiological (A) and pathological (B) architecture of the hepatic sinusoid. The schematic illustrates key features of the hepatic lobule: (i) cellular zonation along the sinusoids; (ii) the presence of an oxygen gradient, which is disturbed in MASLD; and (iii) in MASLD, the predominance of early hepatic lesions in the hypoxic pericentral region. Abbreviations: HSCs, hepatic stellate cells; LAMs, lipid-associated macrophages; LSECs, liver sinusoidal endothelial cells; MASLD, metabolic dysfunction-associated steatotic liver disease. Figure created with BioRender.

The liver performs a range of vital functions, including regulating systemic metabolism, detoxification, plasma protein synthesis, and bile production. To support these processes, the liver exhibits a highly specialised architecture, organised into functional units called lobules (Figure 1). This anatomical structure facilitates directional blood circulation, establishing gradients of hormones, nutrients, and importantly O_2_. Blood enters from the portal vein (~75%) and the hepatic artery (~25%), which, along with the bile ducts, form the portal triad. From there, blood flows and mixes in the highly fenestrated sinusoids and exits through the central vein. Blood from the portal vein is nutrient-rich, carrying sugars and amino acids absorbed from the gastrointestinal tract, while blood from the hepatic artery is O_2_-rich. As a result, higher O_2_ levels occur in the periportal area (partial O_2_ pressure of 60–65 mmHg), while lower levels are found in the pericentral area (partial O_2_ pressure of 30–35 mmHg).

Some studies have measured intrahepatic oxygen levels in humans [4], or at least surrogate markers such as deoxyhaemoglobin [5], but direct measurements in patients with MASLD have not yet been reported. Nevertheless, several studies have demonstrated lower O_2_ levels in MASLD mouse models [6,7]. Consistently, a study using immunostaining for carbonic anhydrase IX (presumably a hypoxia marker) in biopsies from MASLD patients [8] also points to an increased hypoxia in MASLD. Moreover, hepatic fibrotic regions are thought to be hypoxic, and emerging evidence suggests a reciprocal relationship in which hypoxia promotes **fibrosis**, further intensifying local hypoxia and amplifying liver injury [9]. However, the actual contribution of hypoxia in MASLD pathogenesis remains scattered.

A cross-sectional study of 545 liver biopsies of MASLD patients showed that the location of steatosis along the sinusoid, and therefore subjected to different O_2_ levels, was associated with distinct features [10]. It found that steatosis was localised to pericentral hepatocytes in 38% of cases, to periportal hepatocytes in <1%, and to azonal or pan-acinar in 62% [10]; pan-acinar steatosis was associated with hepatocyte ballooning and advanced fibrosis [10]. Similarly, fibrotic lesions tend to arise initially in the pericentral zone and progress outward toward the periportal areas as disease advances [11].

Interestingly, in alcohol-related steatotic disease (ALD), hypoxia appears to be also increased, as shown by oxygen measurements in preclinical models [12]. This is expected because ALD progresses with fibrosis and fat accumulation, which reduces oxygen diffusion as in MASLD, and because alcohol metabolism substantially increases hepatic O_2_ consumption. Notably, similar to MASLD, steatosis in ALD typically begins and is most pronounced in the centrilobular region [13].

The physiological oxygen gradient participates in the metabolic zonation, whereby hepatic cells exhibit distinct functions based on their spatial location within the sinusoid [2,14]. Hepatocyte functional heterogeneity has been recognised for decades [15], but recent advances in spatial single-cell transcriptomics provided insights into zonal gene expression patterns [16,17]. Periportal hepatocytes, exposed to higher O_2_ levels, preferentially undertake processes that rely heavily on oxidative phosphorylation in the mitochondria, such as gluconeogenesis, β-oxidation, and plasma protein synthesis. Conversely, pericentral hepatocytes in more hypoxic regions are enriched for glycolysis, lipogenesis, and xenobiotic metabolism [2,18,19]. They also play a key role in regeneration regulating glutamate levels, which reprogram macrophages toward a pro-regenerative phenotype, thereby enhancing hepatocyte proliferation [20]. Importantly, *in vitro* systems mimicking the hepatic O_2_ partially recapitulate hepatocyte zonation [21,22], thus highlighting the crucial role of O_2_ in establishing liver zonation.

In addition to hepatocytes, hepatic non-parenchymal cells also exhibit spatial zonation [23]. Kupffer cells, the liver-resident macrophages, are more abundant in the periportal region, serving as the first line of defence against pathogens and xenobiotics entering from the gut via the portal vein [24]. However, in MASLD, the hepatic macrophage populations undergo significant remodelling. Monocyte-derived macrophages, recruited in response to injury and inflammation, become the dominant population [25]. The different subtypes of these macrophages also show specific zonation. For instance, lipid-associated macrophages, a lipid-scavenging macrophage subset found in MASLD, are most commonly found in the pericentral zone of human livers [23]. Interestingly, *in vitro* studies have shown that hypoxia reprograms macrophages in several ways, including impaired phagocytic capacity, reduced lysosomal activity, and a shift toward a proinflammatory phenotype [26,27]. Hence, further investigation is required to confirm these effects of hypoxia in the physiological context of the liver.

Other key cell types in the liver are the hepatic stellate cells (HSCs) and the liver sinusoidal endothelial cells (LSECs). According to their spatial localisation along the sinusoid, both HSCs and LSECs exhibit distinct transcriptional, proteomic, and phosphoproteomic profiles [28–30]. Significantly, under pathological conditions, collagen-producing HSCs are most concentrated around the central vein, contributing to pericentral fibrosis [29]. Likewise, LSECs in this region show greater **capillarisation** and loss of endocytic capacity during fibrosis, evidencing a greater dysfunction [30]. Together, these observations suggest that the pericentral zone, the most hypoxic region of the liver, is particularly susceptible to fibrosis. Although *in vitro* studies shows that hypoxia promotes a profibrotic phenotype in HSCs [31,32], it remains elusive whether the *in vivo* vulnerability to fibrosis in this zone is directly driven by low O_2_ levels.

Altogether, these data suggest that pericentral region, characterised by hypoxia, is more susceptible to early lipid accumulation and fibrogenesis. However, more clinical data linking lesion location to outcomes are needed to determine whether zonated assessment has prognostic value (see Clinician’s corner) and future studies may use newly developed tools to bridge this important knowledge gap (Box 1).

### Association between OSA and MASLD

Chronic hypoxia is a shared feature of several clinical conditions linked with metabolic syndrome. Reduced O_2_ levels can result from pulmonary or cardiovascular diseases (e.g., chronic obstructive pulmonary disease, congestive heart failure) (see Box 2 for further information regarding the impact of haemodynamics on liver O_2_ levels), impaired ventilatory control (e.g., **obstructive sleep apnoea, OSA**), or haematologic factors (e.g., anaemia). Within the conditions triggering hypoxia in the liver, OSA is a particularly informative physiological paradigm because it induces acute, recurrent, and well-defined episodes of hypoxia. Notably, OSA provides the most robust and consistent clinical and experimental evidence of a link between chronic intermittent hypoxia (CIH) and the onset and progression of MASLD, with fewer confounding long-term adaptive mechanisms compared with other disorders associated with hypoxia in the liver.

Obesity is a major risk factor for OSA, leading to frequent coexistence of OSA with metabolic syndrome [33], and consequently MASLD. However, multiple studies suggest that OSA contributes to liver injury independently of their shared metabolic risk factors [34–38]. Both in adults [34,35,38] and paediatric patients [37], a positive correlation exists between OSA severity and MASLD severity, remaining significant when adjusting for shared risk factors such as weight and insulin resistance. In a multicentre cohort of 97 patients, OSA was associated with liver fibrosis but not with steatosis or steatohepatitis [38]. By contrast, OSA was linked to multiple MASLD features in a paediatric cohort of 65 children, including the presence of **metabolic dysfunction-associated steatohepatitis (MASH)**, fibrosis, non-alcoholic fatty liver disease (NAFLD) activity score, intrahepatic immune cell infiltration, and circulating markers of hepatocyte apoptosis and fibrogenesis [37]. Finally, a meta-analysis (nine studies comprising 2272 participants) confirmed significant associations between OSA and elevated alanine aminotransferase (ALT) levels, steatosis, lobular inflammation, ballooning degeneration, and fibrosis, but found no correlation with MASLD activity score or aspartate aminotransferase (AST) levels [34]. Overall, the role of OSA as an independent disease amplifier of MASLD within the context of metabolic syndrome is well supported, but the inconsistent associations between OSA and specific histological features of MASLD across studies highlight the need to more precisely identify which components of CIH drive MASLD progression. Mechanistically, this relationship is supported by animal studies showing that CIH exacerbates both hepatic inflammation and fibrosis in diet-induced models of MASLD [39,40].

Despite strong evidence linking CIH to MASLD, correction of hypoxaemia in patients with OSA and MASLD yielded inconclusive results. A meta-analysis restricted to randomised controlled trials (RCTs) concluded that continuous positive airway pressure (CPAP), the standard treatment for OSA, was ineffective in improving hepatic histology or surrogate biomarkers (i.e., serum aminotransferase levels) in MASLD patients [41]. However, these studies suffer from several limitations such as short intervention durations, variable CPAP adherence, small sample sizes, and heterogeneity in MASLD diagnostic criteria. A larger and more rigorously designed RCT involving 120 patients shows that 6 months of CPAP therapy failed to improve any MASLD feature [42]. Lastly, a recent study involving 44 MASLD–OSA patients treated with CPAP for 18 months reported improvements in lipid profile and liver function [43]. Hence, the efficacy of CPAP in improving MASLD remains debatable, and more prolonged and robust clinical trials are needed.

### Hypoxia-sensing mechanisms potentially influencing MASLD

**Figure 2:**
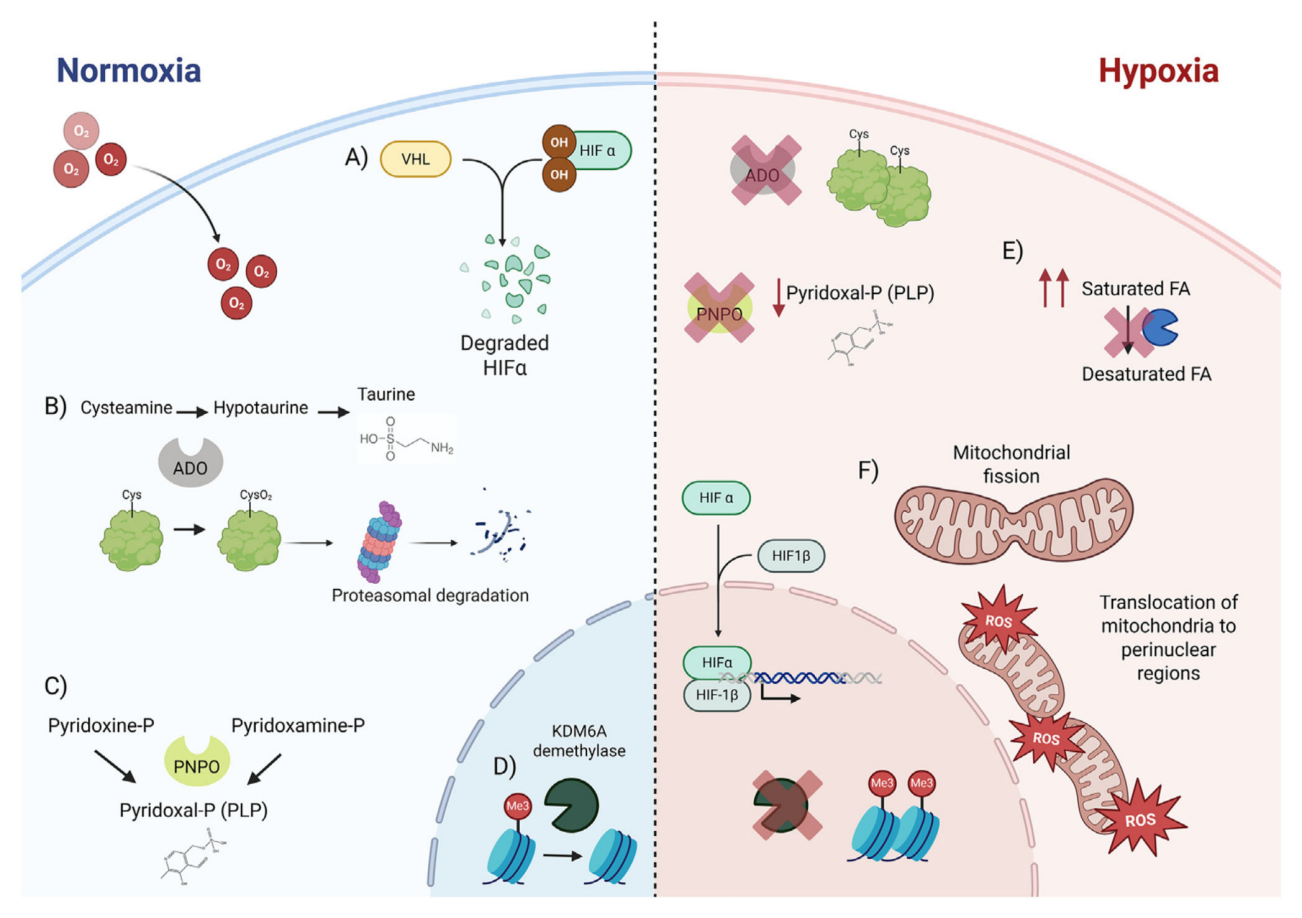
Mechanisms of sensing hypoxia. (A) Hypoxia-inducible factors (HIFs) signalling pathway: in normoxic conditions, HIFs α are degraded, while in hypoxia, they are stabilised and enter the nucleus to activate their target genes. (B) 2-Aminoethanethiol dioxygenase (ADO) mechanism: ADO catalyses the conversion of cysteamine to hypotaurine to synthesise taurine and controls the stability of several proteins by modifying their N-terminal cysteine. This is inhibited in hypoxia. (C) Pyridoxine 5′-phosphate oxidase (PNPO) mechanism: PNPO catalyses the synthesis of pyridoxal-phosphate, a cofactor required for many enzymes, and this reaction only takes place under normoxic conditions. (D) KDM6A histone demethylase mechanism: This histone demethylase is blocked in hypoxia. (E) Lipid homeostasis in hypoxia: during hypoxia, the ratio of saturated to desaturated fatty acids (FAs) is increased, presumably due to inhibition of desaturases. This triggers metabolic adaptations to restore membrane fluidity and other processes dependent on lipid composition. (F) Mitochondrial changes in hypoxia: in response to low levels of O_2_, mitochondrial fission is increased, mitochondria are translocated to the perinuclear regions, and reactive oxygen species (ROS) are induced. Abbreviation: VHL, von Hippel-Lindau Figure created with BioRender.

Cells have molecular mechanisms that enable them to sense and adapt to low oxygen levels. One of the main pathways mediating this response is the well-known hypoxia-inducible factors (HIFs). Although extensively studied, the specific functions of HIFs in chronic liver diseases remain incompletely understood. Beyond HIFs, several HIF-independent hypoxia-sensing pathways have been identified (Figure 2).

#### HIFs in MASLD

HIFs are heterodimers formed by an α-subunit (HIF-1α, HIF-2α, or HIF-3α) and a β-subunit (HIF-1β). While the regulation and function of HIF-1α and HIF-2α are well studied, HIF-3α has been less explored [44]. HIF activity is regulated by oxygen-dependent degradation mechanisms. Under normoxic conditions, HIF-α subunits are hydroxylated by prolyl hydroxylase domain-containing enzymes (PHD1–3), which mark them for ubiquitination and subsequent **proteasomal degradation**. Low levels of O_2_ suppress PHD activity, stabilising HIFs [45], thus allowing their nuclear translocation and transcriptional activity. HIFs trigger metabolic adaptation to hypoxia by promoting glycolysis [46] and suppressing mitochondrial oxidative metabolism, including fatty acid oxidation (FAO), with essential implications for hepatic metabolism [47].

Importantly, HIF stabilisation is not exclusively triggered by hypoxia. Other stimuli can activate or amplify HIF signalling. One of these is the **reactive oxygen species (ROS)** [48], which have been observed to be elevated in mouse models of MASLD [49], but their elevation remains unclear in the liver of MASLD patients [50]. Another mediator that promotes HIF-1α stabilisation is succinate, a key intermediate in the tricarboxylic acid (TCA) cycle [51]. Succinate itself is emerging as a signalling metabolite promoting metabolic reprogramming and fibrosis in liver disease [52]. Additionally, mechanical forces like shear stress or altered extracellular matrix stiffness can activate HIF-1α through mechanotransduction pathways independently of oxygen tension [53]. Furthermore, some studies have shown that gut microbiota-derived metabolites influence HIF stability and activity [54,55], thereby modulating the interplay between the gut and the liver, with important consequences for MASLD [55].

Beyond post-translational regulation, *HIF1A* gene expression is also controlled transcriptionally by various inflammation-activated factors, including nuclear factor-κB (NF-κB), specific protein 1 (SP1), and nuclear factor erythroid 2–related factor 2 (NRF2) [56].

Increased hepatic protein levels of HIFs have been observed in patients with chronic liver disease [57–60] and likely influence several aspects of MASLD pathogenesis [61]. However, HIF activation in MASLD may also arise from hypoxia-independent stimuli, placing HIFs at the intersection of metabolic stress, oxidative damage, mechanotransduction signalling, and inflammatory signalling in the liver, where they may further reinforce pathogenic pathways through self-amplifying feedback loops.

Curiously, in ALD, HIFs also appear to contribute to disease pathogenesis [62,63]. However, hypoxia-mediated responses in ALD may differ from those in MASLD. Notably, a recent study found that CIH decreased HIF-2α in a mouse model of ALD, alleviating hepatic injury [63], which is not the case for MASLD mouse models [39,40].

#### HIFs in hepatic steatosis

The importance of HIFs in liver steatosis was firstly demonstrated in the genetic disruption of the protein that targets HIFs for degradation, von Hippel-Lindau (VHL), which led to constitutive activation of HIFs and severe hepatic steatosis. This phenotype was accompanied with impaired FAO, even in the absence of additional metabolic stressors [64].

Subsequent studies confirmed that hypoxia, through the activation of HIF-2α, promotes hepatic lipid accumulation in both cell-based and animal models [57,60,61,65]. Mechanistically, chronic HIF-2α activation suppresses PPARα signalling, a master regulator of FAO, thereby suppressing lipid catabolism and promoting triglyceride accumulation [65].

By contrast, the role of HIF-1α in steatosis remains controversial and context dependent. In a mouse model of fatty liver based on choline-deficient diet, HIF-1α deletion worsened steatosis [66]. In line with this, in *in vitro* hepatocytes exposed to palmitate, HIF-1α deletion was also exacerbating the lipotoxicity [67]. Conversely, in diet-induced MASLD, antisense oligonucleotide-mediated HIF-1α knockdown in the liver and adipose tissue attenuated steatosis [68].

The metabolic effects of HIF activation extend beyond lipid handling, intersecting with insulin signalling and glucose homeostasis. In both the hyperphagic db/db mouse and in high-fat diet (HFD)-induced MASLD, promoting HIF-2α activation improved insulin sensitivity by directly up-regulating Irs2, a key component of the insulin signalling cascade [69,70]. However, excessive HIF-2α activation, achieved through deletion of all three PHD isoforms (Phd1–3), aggravated hepatic steatosis [70]. Therefore, overall, moderate HIF-2α activation appears to be beneficial for insulin sensitivity and glucose handling, while HIF-2α overactivation promotes steatosis, high-lighting the challenge of fine-tuning HIF-2α in MASLD.

Regarding HIF-1α, hepatocyte-specific deletion of *Hif1a* impaired glucose tolerance and insulin sensitivity in HFD-induced MASLD [71]. However, another study using a different nutritional model of obesity found that treatment with HIF-1α antisense oligonucleotides improved glucose and insulin levels [68]. These seemingly contradictory findings suggest that HIF-1α may exert distinct effects depending on tissue specificity or disease stage.

#### HIFs in hepatic inflammation

Hypoxia, and HIF-mediated pathways in particular, have a significant impact on immune cell metabolism and, consequently, on their activity. This topic has been reviewed extensively recently [72]. Therefore, the focus here will be exclusively on MASLD.

In macrophages, which play a pivotal role in orchestrating hepatic inflammation in MASLD, HIF-1α and HIF-2α regulate distinct gene programs [73–75].

Constitutive activation of HIF-1α in myeloid cells exacerbated MASH induced by a choline-deficient diet. This effect was attributed to impaired autophagic flux in macrophages, promoting their proinflammatory overactivation [76].

However, myeloid-specific deletion of HIF-2α in a HFD model conferred protection against MASLD. HIF-2α promoted Kupffer cell death and proinflammatory activation in the recruited hepatic macrophages [77], thus enhancing liver inflammation. Moreover, hepatocyte-specific deletion of HIF-2α ameliorated MASH through the reduced secretion of proinflammatory histidine-rich glycoprotein (HRGP) in a methionine/choline-deficient diet mouse model [57]. Furthermore, a HIF-2α-specific inhibitor reduced inflammatory gene expression in human liver spheroids cultured under MASH-like conditions, pointing to an overall beneficial anti-inflammatory effect [77].

#### HIFs in hepatic fibrosis

Increased HIF-1α expression has been consistently observed in preclinical models of liver fibrosis as well as in cirrhotic patients [59]. In mouse models of MASLD, *Hif1a* deletion in hepatocytes reduced fibrosis, supporting its profibrotic role [79–81]. Mechanistically, HIF-1α promotes the up-regulation of profibrotic factors in hypoxic hepatocytes [82,83] and macrophages [84,85], and the profibrotic phenotype of HSCs [86–88]. However, the role of HIF-2α in hepatic fibrosis remains incompletely defined. *In vivo* inhibition of HIF-2α reduced hepatic fibrosis [77], but these effects could be secondary to its beneficial effects on MASLD. Similarly, hepatocyte-specific HIF-2α deletion alleviated fibrosis in a CCl -induced fibrosis model, potentially reflecting reduced hepatocyte death rather than a direct effect on fibrogenesis [89]. Therefore, further studies are needed to investigate whether HIF-2α is a direct contributor to fibrogenesis.

#### HIF-independent mechanisms for hypoxia sensing

A particularly intriguing insight into HIF-independent hypoxia sensing emerged from a genome-wide clustered regularly interspaced short palindromic repeats (CRISPR) screen, which identified 322 genes essential for cellular adaptation to hypoxia. Remarkably, the majority of these genes were unrelated to HIF signalling, but instead mapped to pathways associated with mitochondrial function, lipid metabolism, and peroxisomal activity [90]. Hypothesised by the authors of this paper and supported by other studies [91,92], hypoxia inhibits the activity of the lipid desaturases leading to an increased in the saturated fatty acids (SFA). This is biologically plausible, as desaturases require molecular O_2_ as an electron donor. Notably, an increased SFA-to-unsaturated fatty acid (UFA) ratio promotes endoplasmic reticulum (ER) stress [93] and proinflammatory signalling pathways [94] in steatotic livers. Thus, lipid desaturases may serve as metabolic regulators that respond to O_2_ availability, shaping lipid composition and modulating hepatic stress. In fact, the unsaturation degree of lipids has been shown to be decreased in hepatic fibrosis around the central vein, the most hypoxic area of the sinusoid [95].

Hypoxia also triggers HIF-independent mitochondrial adaptations that are highly relevant to MASLD such as mitochondrial fission [96]. Increased mitochondrial fission has been observed in the liver of HFD-fed mice, and promoting mitochondrial fusion improved hepatic steatosis [97].

In addition, hypoxia increases mitochondrial ROS production at complex III, promoting mitochondrial translocation toward the nucleus, where they facilitate redox signalling that modulates gene expression and stress responses [98]. Hypoxia-induced ROS also activate AMP-activated protein kinase (AMPK), a key energy sensor that limits ATP-consuming biosynthetic pathways and downregulates O_2_-demanding processes, thereby enhancing cell survival under hypoxic conditions [99,100]. Elevated mitochondrial ROS are a hallmark of MASLD and may, at least in part, arise secondary to hypoxia. However, some studies report higher ROS marker levels in the periportal than in the pericentral region [101]. Excess ROS lead to oxidative stress, lipid peroxidation, and hepatocellular injury, thereby fuelling hepatic inflammation and disease progression [102].

A recently proposed O_2_ sensor is the histone demethylase KDM6A. Under hypoxic conditions, KDM6A activity is inhibited, leading to increased **histone methylation** and thereby modifying the chromatin states [103]. While KDM6A demethylase is expressed in hepatic cells (https://www.livercellatlas.org/), its physiological relevance in liver function and disease remains to be established. Nevertheless, epigenetic regulation through histone methylation is increasingly recognised as a contributor to MASLD progression, and preclinical studies show that modulating histone methylation can ameliorate MASLD, highlighting it as a potential therapeutic strategy [104].

Another described O_2_-sensing mechanism involves 2-aminoethanethiol dioxygenase (ADO) [105], an enzyme that operates within a similar oxygen range as the PHD/HIF pathway [106]. Beyond its canonical role in sulfur amino acid metabolism, ADO regulates protein stability through oxidation of N-terminal cysteine residues, marking them for proteasomal degradation via the N-degron pathway. Of potential relevance in MASLD, ADO targets include G protein signalling regulators 4 and 5 (RGS4 and RGS5) and interleukin-32, which are implicated in cell proliferation and inflammation [107]. Although ADO is expressed in hepatocytes (https://www.livercellatlas.org/), its physiological role in the liver, and potential contribution to MASLD, is still largely unexplored.

Finally, pyridoxine 5′-phosphate oxidase (PNPO) has recently been identified as an O_2_-sensing enzyme [26]. PNPO catalyses the conversion of pyridoxine 5′-phosphate to pyridoxal 5′-phosphate (PLP), the active cofactor form of vitamin B6, through a strictly O_2_-dependent reaction. In macrophages, chronic hypoxia reduces PLP production, impairing lysosomal function and promoting a proinflammatory phenotype [26]. Whether this mechanism occurs in hepatic macrophages or other liver cell types and contributes to zonated inflammation in MASLD remains an open question.

### Concluding remarks

**Figure 3:**
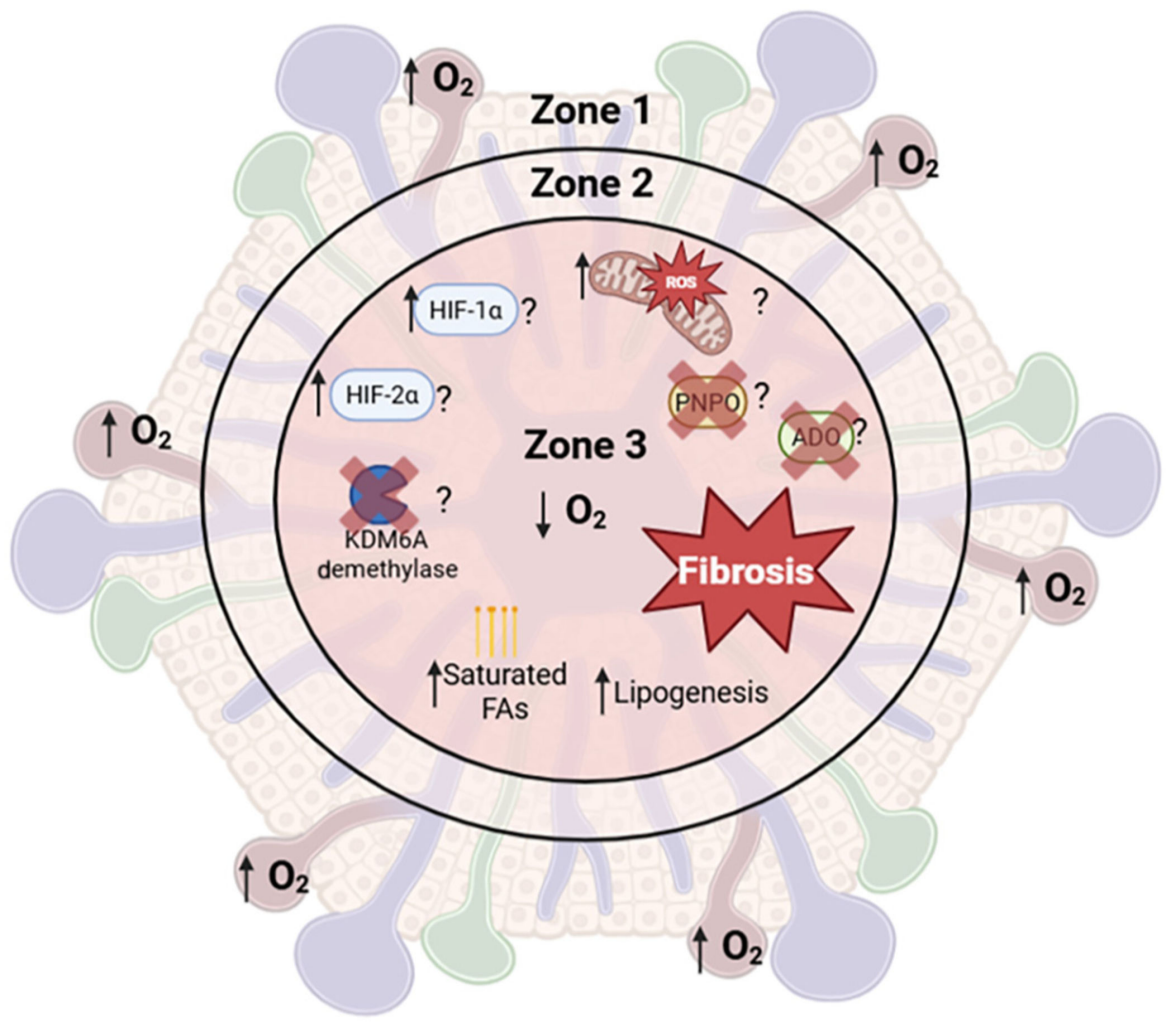
Schematic representation of proposed oxygen-sensing mechanisms in the hepatic lobule and their potential contribution to the zonated vulnerability of the pericentral region. Zone 3 of the lobule represents the most hypoxic area, where hepatocytes preferentially perform lipogenesis rather than β-oxidation, the degree of lipid saturation is increased, and fibrotic lesions typically arise; all of which are key processes in MASLD pathogenesis. Further investigation of these oxygen-sensing pathways is required to clarify their location in the pericentral region and their role in the development of MASLD. Abbreviations: ADO, aminoethanethiol dioxygenase; FA, fatty acid; MASLD, metabolic dysfunction-associated steatotic liver disease; PNPO, pyridoxine 5′-phosphate oxidase. Figure created with BioRender.

Growing evidence implicates hypoxia and O_2_-sensing pathways as central regulators of MASLD pathogenesis. Beyond the well-characterised role of HIF signalling in MASLD, HIF-independent hypoxia responses, including mitochondrial dysfunction, metabolic rewiring, redox imbalance, and epigenetic regulation, may represent underappreciated contributors to disease progression (Figure 3). Notably, targeting some hypoxia-linked mechanisms (i.e., HIF-2α antagonists, mitochondrial-directed therapies, and redox modulators) has shown promise in preclinical studies.

Despite progress in defining hypoxia-responsive signalling, how spatial and temporal variations in hepatic oxygenation influence disease initiation and progression remain poorly understood (see Outstanding questions). A significant challenge is the limited integration of direct tissue O_2_ measurements into experimental and clinical MASLD studies. Mapping of intrahepatic O_2_ gradients could clarify whether altered sinusoidal oxygenation disrupts metabolic zonation and promotes inflammation and fibrosis during disease progression. Indeed, a key unresolved question is whether hypoxia acts as an early pathogenic driver and how oxygenation dynamics evolve over the course of disease.

In summary, a critical next step for the MASLD field is to address how hypoxia is sensed, integrated, and organised across spatial and temporal scales within the liver, as this may enable more precise disease classification and inform therapeutic intervention (see Clinician’s corner).

## Glossary

**Capillarisation:** transformation of fenestrated, discontinuous sinusoidal endothelial cells into a continuous, capillary-like endothelium is characterised by the loss of fenestrations, the deposition of a basement membrane, and reduced permeability.

**Cirrhosis:** end stage of chronic liver disease, characterised by extensive fibrosis and disruption to the liver’s architecture. These changes lead to portal hypertension and hepatic insufficiency. Cirrhosis substantially increases the risk of hepatocellular carcinoma and liver-related mortality.

**Fibrosis:** biological process characterised by the accumulation of extracellular matrix components, primarily collagen, as a wound-healing response to injury. It results in architectural distortion and impaired function of the organ in which it is presented.

**Histone methylation:** modification on histone proteins involving the addition of methyl groups to specific lysine or arginine residues, which serve as the structural core around which DNA is wrapped. It acts as an epigenetic mechanism regulating chromatin structure and gene expression by influencing the accessibility of transcriptional machinery to DNA.

**Metabolic dysfunction-associated steatohepatitis (MASH):** stage of MASLD, in which there is an evident inflammatory state. Histologically, it is defined by the presence of hepatocellular ballooning, lobular inflammation, and varying degrees of fibrosis, in addition to steatosis.

**Metabolic dysfunction-associated steatotic liver disease (MASLD):** chronic liver disease characterised by excessive lipid accumulation within hepatocytes. It occurs in the context of metabolic dysfunction, such as obesity, insulin resistance, dyslipidaemia, or type 2 diabetes. It encompasses a spectrum of hepatic alterations ranging from simple steatosis to inflammation and fibrosis. MASLD is the most prevalent chronic liver condition, affecting over 30% of adults worldwide, and ranking among the leading causes of liver transplantation and liver-related mortality. The term MASLD reflects an updated diagnostic criteria of NAFLD that emphasise metabolic dysfunction. However, the differences between NAFLD and MASLD are minimal and this review adopts the term MASLD for both terminologies. While many individuals exhibit simple steatosis with an apparently benign course, a non-negligible subset of patients progresses to MASH, which markedly increases the risk of cirrhosis and hepatocellular carcinoma.

**Obstructive sleep apnoea (OSA):** common sleep-related breathing disorder characterised by repeated episodes of partial or complete upper airway obstruction during sleep, causing cycles of apnoea and hypopnea that are typically followed by brief arousals to restore normal breathing.

**Proteasomal degradation:** cellular process that is responsible for the selective breakdown of specific proteins. It involves the ubiquitin–proteasome system, whereby proteins are tagged with ubiquitin, allowing them to be recognised and degraded by the proteasome.

**Reactive oxygen species (ROS):** highly reactive oxygen-containing molecules, such as superoxide anion ${\text{(O}}_{2}^{-})$, hydrogen peroxide (H_2_O_2_), and hydroxyl radicals (•OH). They can be produced as by-products of cellular metabolism, particularly within mitochondria. While low levels of ROS play a signalling role, excessive generation of ROS leads to oxidative stress, which damages lipids, proteins, and DNA. This contributes to inflammation and cell death in liver diseases such as MASLD.

## Box 1. How to measure intrahepatic O2 levels?

The recognition of the role of localised hypoxia in various pathologies, including cancer, is increasing alongside the development of methods to assess oxygen levels within organs. However, these approaches remain limited in scope, with each offering distinct advantages, limitations, and varying feasibility for application in human studies.

Direct measurements using O_2_-sensitive optical nanosensors provide quantitative partial O_2_ pressure readings with high spatial resolution, enabling the assessment of zonal oxygen gradients. Nevertheless, these techniques are highly invasive and only sample localised regions, which restricts their use primarily to animal models or *ex vivo* human tissue [108,109].

In addition, there are techniques based on magnetic resonance imaging (MRI) such as blood oxygen level-dependent, that detect deoxyhaemoglobin to infer tissue oxygenation [5]. While these methods are suitable for human studies, they provide indirect measures of oxygenation, are technically demanding, and offer limited spatial resolution.

Finally, indirect markers of hypoxia, including pimonidazole adducts (which identify regions with partial O_2_ pressure below ~10 mmHg) and molecular responses such as HIF stabilisation or carbonic anhydrase IX, can be assessed using histology and immunostaining. While these markers can be useful for identifying hypoxic regions and down-stream responses, they do not provide quantitative or dynamic measurements of tissue O_2_ levels. Therefore, these measurements require cautious interpretation given their methodological limitations. Also, recent advances in spatial multi-omics offer new powerful avenues for the indirect detection of molecular signatures associated with O_2_ consumption and its regional variations. High-resolution imaging-based spatial transcriptomics platforms can map the expression of genes involved in high-oxygen-demanding processes while simultaneously identifying hypoxia-responsive programs in the pericentral area. Furthermore, MALDI mass spectrometry imaging (MALDI-MSI) enables the label-free spatial mapping of metabolites that serve as functional surrogates for O_2_ availability; for example, the ratio of SFA to UFA indicating local O tension.

## Box 2. Why do haemodynamics matter for intrahepatic oxygenation?

The importance of haemodynamics for hepatic oxygenation is clearly demonstrated in heart failure. In acute heart failure, for example, reduced cardiac output limits hepatic perfusion, leading to insufficient O_2_ delivery and, therefore, ischaemic liver injury. In chronic heart failure, elevated central venous pressure causes passive hepatic congestion, impairing sinusoidal blood flow and reducing effective O_2_ diffusion to hepatocytes. This condition, known as congestive hepatopathy, primarily affects the centrilobular region, exacerbating the localised hypoxia of this hepatic region. Persistent congestion promotes fibrosis, which begins in the centrilobular zone and progressively extends towards the portal areas. This further compromises microvascular perfusion and O_2_ exchange. While hepatic injury may be reversible with an improvement in cardiac function, prolonged hypoxia and congestion in advanced heart failure can lead to irreversible fibrosis and the development of **cirrhosis** [110].

In addition, portal hypertension may compromise the delivery of O_2_ to hepatic cells via several interconnected mechanisms. Increased intrahepatic vascular resistance and architectural distortion reduce the effective perfusion of the hepatic sinusoids by the portal vein. Moreover, sinusoidal capillarisation, which is associated with portal hypertension and is characterised by loss of permeability, impairs microvascular perfusion and thereby is expected to limit the diffusion of oxygen from the sinusoidal blood to the parenchyma. Meanwhile, intrahepatic hypoxia can also influence sinusoidal pressure, as reduced O_2_ availability promotes intrahepatic angiogenesis via several growth factors such as vascular endothelial growth factor (VEGF) and platelet-derived growth factor (PDGF). In cirrhosis, angiogenesis initially represents an adaptive response to hypoxia, aimed at improving O_2_ and nutrient delivery in capillarised regions. However, excessive and dysregulated angiogenesis in advanced disease contributes to increased intrahepatic resistance and further aggravates portal hypertension. Consistent with this concept, preclinical studies have shown that targeting angiogenic pathways (i.e., inhibiting PDGF signalling or blocking VEGF receptors with agents) can reduce portal pressure, hepatic fibrosis, and inflammation in experimental models of cirrhosis [111].

## Clinician’s corner

Oxygen (O_2_) gradients are disrupted in MASLD, potentially influencing the course of disease progression. Although lipid accumulation may occur in various zones of the hepatic sinusoid, it most frequently develops in the pericentral zone, where O_2_ levels are lowest. Likewise, fibrosis in MASLD typically originates near the central vein.

Only a few studies have examined whether these spatial patterns reflect distinct pathogenic mechanisms. However, preclinical evidence shows that oxygen levels modulate cellular responses in hepatocytes, hepatic stellate cells, and macrophages, suggesting that the architecture of the hepatic sinusoid modulates MASLD progression.

Given the crucial role of O_2_ availability in regulating inflammatory and fibrotic responses, evaluating hepatic oxygenation in MASLD could provide valuable insights into the disease. Incorporating oxygen gradient to the spatial dimension of hepatic organisation is essential for a comprehensive understanding of disease pathophysiology.

Mapping O_2_ gradients and cellular states along the sinusoid, and linking these to clinical outcomes, is emerging as a key research topic in the quest to better understand MASLD pathophysiology. Current diagnostic approaches remain limited in their ability to capture spatial and intrahepatic O_2_ levels. With the development of tools to estimate intrahepatic O_2_ distribution, an O_2_-informed view of the liver could improve patient classification, refine prognostic assessment, and guide personalised therapy.

Targeting hypoxia-related pathways presents novel therapeutic opportunities. In preclinical models, HIF-2α antagonists have demonstrated promising efficacy. Although the effectiveness of CPAP in improving MASLD remains unclear, preclinical data strongly supports the integration of hypoxia management into multimodal care rather than treating it as an isolated comorbidity. For that reason, actively screening for OSA as well as assessing its severity in MASLD patients might guide risk stratification and targeted therapies.

Overall, although zonated hypoxia in MASLD requires further clarification, a deeper understanding of O_2_-driven mechanisms may help to bridge the gap between preclinical promises and clinical successes.

## Outstanding questions

How do oxygen levels shape liver zonation?

Can disrupted oxygen flow drive the zonal differences in hepatic lesions during MASLD?

Does the location of the initial hepatic lesions along the O_2_ gradient impact MASLD progression?

To what extent do oxygen levels modulate macrophage and hepatic stellate cell behaviour in the physiological context of MASLD?

Beyond HIFs, which are the key molecular mediators of the hypoxic response in the liver?

Can zonation-based biomarkers or noninvasive imaging reliably reflect hepatic oxygenation in MASLD patients?

How does hepatic hypoxia evolve across MASLD stages, and what aspects of disease progression does it reflect or drive?

Do hepatic oxygenation dynamics hold prognostic value in MASLD?

Should therapeutic interventions target specific hepatic regions, particularly hypoxic zones, for more effective MASLD treatment?

## Highlights

Oxygen gradient drives the liver’s zonated architecture, with metabolic dysfunction-associated steatotic liver disease (MASLD) lesions preferentially emerging in the hypoxic pericentral region.

In MASLD, architectural distortion and fibrosis intensify hypoxia, influencing liver injury, as oxygen levels serve as a spatial determinant of the hepatic response to injury.

In patients with metabolic syndrome, obstructive sleep apnoea leads to chronic intermittent hypoxia, exacerbating MASLD progression.

Canonical [hypoxia-inducible factor (HIF)-dependent] and emerging HIF-independent oxygen-sensing pathways converge on lipid metabolism, inflammation, and fibrogenesis, implying that hypoxia shapes liver injury in MASLD.

Spatial omics will improve our understanding of the molecular responses to hypoxia in MASLD, which may enable better prognostication and spatially informed therapies.
